# Foley catheter for cervical preparation prior to second trimester dilation and evacuation: A supply-based alternative for surgical abortion: A case series

**DOI:** 10.1016/j.conx.2022.100085

**Published:** 2022-10-13

**Authors:** Abraham Fessehaye Sium, Sarah Prager, Mekdes Wolderufael, Ferid A. Abubeker, Tesfaye H. Tufa, Jaclyn M. Grentzer

**Affiliations:** a Department of Obstetrics and Gynecology, St.Paul's Hospital Millennium Medical College (SPHMMC), Addis Ababa, Ethiopia; b Family Planning Division, Department of Obstetrics and Gynecology, UW Medicine, Seattle, WA, United States

**Keywords:** D&E, Foley catheter, Surgical abortion, Second trimester abortion, Intra-cervical Foley catheter placement, Cervical preparation

## Abstract

**Objective:**

To document the use of Foley catheter as an alternative to osmotic dilators for cervical preparation before second trimester dilation and evacuation at a tertiary setting in Ethiopia

**Methods:**

This is a retrospective case series conducted at St. Paul's Hospital Millennium Medical College (Ethiopia) from April 1, 2021 to August 30, 2021. Forty-three cases of dilation and evacuation (D&E) in which overnight intra-cervical Foley catheter placement was used to prepare the cervix were reviewed. Data were analysed using SPSS version 23 and simple descriptive statistics were applied to analyze the clinical characteristics of study subjects and procedure outcomes. Percentages and frequencies were used to present the findings.

**Results:**

The calculated median gestational age was 21.4(17–24.2) weeks. Around 40%(17/43) of cases had cervical dilation of 3 to 4 cm, with another 33% (14/43) achieving cervical dilation of 1.5 to 2.5 cm. Additional dilation during the procedure was required only in 12 cases (28%) prior to D&E procedure. In 36 cases (84%), the procedure was attended by family planning fellow.

**Conclusion:**

Cervical preparation using overnight Foley catheter before second-trimester D&E resulted in majority of the cases requiring no additional dilation immediately prior to D&E procedure. Where osmotic dilators are not available, Foley catheter can be an alternative method to prepare the cervix prior to D&E procedures.

**Implications:**

In the absence of osmotic dilators, Foley catheter can be used as an alternate, and readily available means of cervical preparation for second trimester D&E procedures

## 1 Introduction

Abortion during the second trimester of pregnancy accounts for 10% to 15% of abortions performed worldwide [1], [2]. Dilation and evacuation (D&E) has become the preferred method of second-trimester abortion for it's relative saftey. In United States it accounts for 95% second trimester abortion procedures [3], [4]. Complications from D&E increase with advancing gestational age and include cervical laceration (0.9%), infection (0.4%–2%), hemorrhage (up to 10 in 1000), and uterine perforation (0.1–3 in 1000) [5], [6], [7]. Cervical preparation has been shown to decrease the risk associated with D&E. Although there is no standard approach to cervical preparation, evidence-based options that are safe and effective include misoprostol, mifepristone, osmotic dilators, and combinations [8]. The 2022 WHO Abortion Care guideline recommends the use of combined method of cervical preparation (osmotic dilators plus mifepristone or misoprostol or the combination of both) before D&E at ≥19 weeks of gestation. This recent guideline also suggests the use of either combined cervical preparation or osmotic dilators alone or medical cervical preparation, preferably mifepristone plus misoprostol prior to D&E at ≥12<19 weeks [9].

Intra-cervical Foley catheter placement has been used for cervical preparation during induction of labor. Multiple studies have demonstrated its efficacy in cervical preparationwith an advantage of cheaper cost, compared to pharmacologic methods [10]. The mode of action for Foley catheter is postulated to be a direct mechanical effect or release of prostaglandins secondary to separation of the gestational sac membranes [11].

There are too few studies on it's practice and safety prior to second trimester D&E. One earlier study compared 25 second trimester cases that had cervical preparation with Foley catheter with 25 controls that had cervical preparation with a 3 mg dinoprostone pessary and concluded that Foley catheter is a readily available and efficacious means of cervical preparation [12]. Similar findings has been documented in another recent study in which 290 cases of D&E at 18 to 21 weeks of gestation, with misoprostol plus same-day Foley catheter used to prepare the cervix were retrospectively reviewed. In the study, the median time between Foley placement and first misoprostol dose and the procedure start was 7.2 hours (2.9–12.6 hours; interquartile range [IQR] 6.2–8.4 hours) with two doses of misoprostol required in most cases [13].

In Ethiopia, following the launch of the family planning fellowship program at St.Paul's Hospital Millennium Medical College, second trimester D&E has been added to the list of clinical services that are provided at this tertiary Hospital. After the introduction of D&E, a retrospective study was published demonstrating the effectiveness of D&E procedure in the setting [14]. In 2021, because of temporary supply shortages of osmotic dilators we switched from osmotic dilators to Foley catheter for cervical preparation before D&E. The aim of this study was to document the practice and safety of using Foley catheter as an alternative to osmotic dilators for cervical preparation before second trimester D&E at our hospital.

## 2 Methods

This is a retrospective cases series conducted at St. Paul's Hospital Millennium Medical College (Addis Ababa, Ethiopia) from April 1, 2021 to August 30, 2021. The Hospital is one of the leading tertiary Hospitals in Ethiopia.

We included 43 patients in this case series who presented for D&E from 17 to 24 weeks and had a Foley catheter for cervical preparation. We included minors and patients diagnosed with fetal anomalies. We excluded patients presenting with fetal demise. Patients presented to the outpatient clinic the day prior to the procedure and gestational age was confirmed on ultrasound. We do not use induced fetal demise for D&E patients. The day prior to the procedure a vaginal speculum was placed, and the cervix was prepped with povidine-iodine. The Foley catheter was placed under direct visualization using ring forceps to guide the tip of the catheter into the cervical os. The Foley catheter balloon was inflated to 30 to 50 mL of normal saline and taped to the patient's leg. The patient was either admitted or discharged home as well as mifepristone 200 mg orally for cervical preparation overnight. Admission or discharge was primarily based on social factors and geographic distance to the patient's home. The next day, patients were given misoprostol 400 µgsublingually and doxycycline 200 mg orally for antibiotic prophylaxis, two hours and half-hour prior to the start of D&E procedure respectively. After two hours, the cervix was checked using a digital pelvic exam to assess dilation. If dilation was found to be adequate, the balloon was deflated, and the patient transferred to the operating room. Adequate dilation was defined as 1.5 cm for patients 17 to 18 weeks, 2 cm for patients 18 to 20 weeks, and 2.5 to 3 cm for patients 20 to 23 weeks. If dilation was not adequate, another dose of misoprostol was given and repeated every two hours until adequate dilation was reached. D&E procedures were performed in the operating room by family planning fellows under the supervision of attending physician(family planning specialist) using either IV moderate sedation with a paracervical block, spinal anesthesia, or deep sedation. In some cases, the procedure was attended by the attending physician

Data were collected by family planning fellows electronically using a data extraction form. The primary outcome was the proportion of patients who had adequate cervical dilation (that is, needing no further mechanical dilation prior to the procedure). Other outcomes were cervical laceration defined as a laceration requiring suturing, post-abortal hemorrhage, and extramural expulsion (expulsion before starting the D&E procedure). Being a retrospective study, informed consent was not needed,so not obtained. Data was entered into SPSS version 23 and simple descriptive statistics were used for analysis. Results are presented as percentages and frequencies. A formal letter of ethical clearance was obtained from the IRB at St.Paul's Hospital Millennium Medical College for this study.

## 3 Results

In this study, 43 cases were reviewed. The median age was 23 years (Table 1). Three subjects (7%) had a history of prior uterine scar, and eight (19%) had prior vaginal deliveries. The calculated median gestational age was 21.4 (17–24.2) weeks. An 18 Fr sized Foley catheter was used in the majority of cases, 39/43 (91%) (Table 2), and in 40/43 (93%) cases Foley balloon inflation volume was 50 mL of normal saline. The most commonly used mode of anesthesia was spinal, represented in 20/43(47%) of the cases. Five subjects went home overnight after placement of the intracervical catheter, while the rest (88%) were admitted overnight after Foley catheter insertion. Thirty-six cases (84%) out of the total were attended by FP fellows.

**Table 1:** Reproductive characteristics of second trimester D&E with Foley cather for cervical preparation in Ethiopia in 2021 (*n* = 43).

Variable	Category	*n*	%
Age	Median	23 y	23%
Gravidity	Primigravida	33	77%
	Multigravida	10	23%
Any prior uterine scar	No	40	93%
	Yes	3	7%
Any prior vaginal delivery	No	35	81%
	Yes	8	19%
Gestational age (in weeks)	Median	21.4 (17–24.2)	
	22–24 wk	18	42%
	20–21 wk	12	28%
	16–19	13	30%

**Table 2:** Overnight intracervical Foley catheter placement for cervical preparation prior D&E - intervention characteristics-in Ethiopia in 2021 (*n* = 43).

Variable	Category	*n*	%
Foley catheter size	16 Fr	1	2%
	18 Fr	39	91%
	20 Fr	2	5%
	24 Fr	1	2%
Foley inflation volume	30–45 mL	3	7%
	50 mL	40	93%
Overnight admission	No	5	12%
	yes	38	88%
Anesthesia	no anesthesia provided	7	16%
	Sedation	15	35%
	Spinal	20	47%
	Spinal + sedation	1	2%
Family planning provided	Nexplanon	28	65%
	Not documented	5	12%
	DMPA	4	9%
	Declined	4	9%
	IUCD	2	5%
Care provider in charge	Attending physician	7	16%
	FP Fellow	36	84%

Most subjects, 36/43(84%) received ≤ 2 doses of misoprostol (Table 3). In 17 cases (40%) from the total a cervical dilation of 3 to 4 cm was attained, followed by cervical dilation of 1.5 to 2.5 cm in another 14 cases (33%). Thirty-one cases (72%) required no additional mechanical dilation at the time of the D&E. The rest had dilation with Pratt dilators in the range of 55 to 77 Fr. Among the complications observed, there were four cervical laceration which required suturing to arrest bleeding, accounting for 9%. All the lacerations were encountered in subjects with an adequate cervical dilation per corresponding gestational age as declared by the family planning fellows who attended those procedures. There were six extramural complete expulsions before D&E, which comprises 14%. There was no encounter of post-abortal hemorrage. We didn't follow patients for delayed complication, hence it was not analyzed.

**Table 3:** Second trimester dilation and evacuation (D&E) with Foley catheter for cervical preparation- procedure outcomes- in Ethiopia in 2021 (*n* = 43).

Variable	Category	*n*	%
Doses of misoprostol provided	No misoprostol	2	5%
	1 or 2	36	84%
	3	3	7%
	4	2	5%
Cervical dilation achieved (cm)	1	3	7%
	1.5–2.5	14	33%
	3–4	17	40%
	>4	3	7%
	No cervical dilation	1	2%
	Not documented	5	12%
Need for mechanical dilation	No	31	72%
	Yes (55–77 Fr)	12	28%
Complications	No complication	29	67%
	Cervical laceration	4	9%
	Expelled before D&E procedure	6	14%
	No cervical dilation achieved (Shifted to medication abortion)	1	2%

## 4 Discussion

In this retrospective case series, adequate cervical dilation was achieved in majority of the cases after overnight intracervical Foley catheter placement. Additional mechanical dilation was required in only 12 cases (28% of the total). Cervical laceration was encountered in four cases, all in patients with adequate dilation. There were six cases of extramural delivery and one case shifted to medication abortion as no cervical dilation was achieved.

The requirement for additional mechanical dilation immediately before D&E procedures in our study is comparable to 26 % reported in a recent study when the cervix was prepared with osmotic dilators at gestational age of 16-23 6/7 weeks, but higher than a rate of 9% found in the same study when both osmotic dilators and adjuvant misoprostol were used to prepare the cervix [15]. In the present case series, adjuvant misoprostol was used in most cases, 36/43(84%) receiving 1 to 2 doses prior to their D&E.

In the last several years multiple randomized trials have evaluated methods of cervical preparation before D&E abortion. The results of these trials have led to improvements in cervical preparation [16]. While laminaria and other osmotic dilators have been established as safe and effective ways to dilate the cervix [17,18], this study provides some evidence for adding Foley catheter as another method of cervical preparation.

Cervical laceration with possible resultant hemorrhage is one of the most cited serious D&E complications [19,20]. The introduction of osmotic laminaria tents dramatically decreased the incidence of cervical laceration requiring repair for procedures between 20 and 26 weeks from 10% to 1% [5]. This study reports 4 cases of cervical laceration, which represents 9%. This finding is comparable to a cervical laceration rate of 9% reported by Poons and Parsons in their review of 34 D&Es with cervical preparation using Dilapan osmotic dilators with or without misoprostol [21] and much higher than a 3% rate reported in a recent randomized controlled trial where osmotic dilators were used to prepare the cervix [15]. A possible explanation for this high rate of cervical laceration in our case series could be the fact that more than 80% (36/43) of the cases were attended by less experienced first year family planning fellows and all the cervical laceration cases were attended by these fellows. The other explanation is advanced gestational age (all the cases were at gestational age greater than 21 weeks). However, it is also possible that the technique (Foley cather as method of cervical preparation) affects the cervix in a way that heightens the chances of clinically significant lacerations.

The strengths of this study are the ability to measure cervical dilation in an objective way that corresponds to the level of dilation required to proceed with D&E procedure at corresponding specific gestational ages, and analysis of procedure related complications, which is an important element of measuring outcomes of an intervention. The main limitations of this study are small sample size and the retrospective nature of the study. A prospective comparative analytic study (Foley group vs osmotic dilators group), preferably a randomized controlled trial would have generated stronger results but was not feasible for many reasons, including the scarcity of osmotic dilators and the urgent need to find a method of cervical preparation. The other limitation of this study is a shorter study period (from April 1, 2021 to August 30, 2021). However, it was only during this time period Foley cather was used routinely as a method of cerivical preparation due to inadequate supply of Laminaria at the study setting.

In summary**,** our study shows that cervical preparation using Foley catheter before second trimester D&E is safe intervention with an acceptable rate of complications. In the absence of osmotic dilators, especially in low-income countries where osmotic dilators are often financially or otherwise unavailable, Foley catheter can be used as an alternate, and readily available means of cervical preparation for second trimester D&E procedures. We recommend a further prospective comparative study inclusive of analysis delayed complications and client/patient acceptance of the method and satisfaction.
